# Commonalities and Specificities in Wheat (*Triticum aestivum* L.) Responses to Aluminum Toxicity and Low Phosphorus Revealed by Transcriptomics and Targeted Metabolomics

**DOI:** 10.3390/ijms25179273

**Published:** 2024-08-27

**Authors:** Daozhen Luo, Qing Li, Fei Pang, Wenjie Zhang, Yangrui Li, Yongxiu Xing, Dengfeng Dong

**Affiliations:** 1 Guangxi Key Laboratory of Agro-Environment and Agric-Products Safety, College of Agriculture, Guangxi University, Nanning 530004, China; 2117303008@st.gxu.edu.cn (D.L.); 2217303006@st.gxu.edu.cn (Q.L.); 2317401006@st.gxu.edu.cn (F.P.); 2117401013@st.gxu.edu.cn (W.Z.); xyx@gxu.edu.cn (Y.X.); 2 Sugarcane Research Institute, Guangxi Academy of Agricultural Sciences, Nanning 530007, China; liyr@gxaas.net

**Keywords:** wheat root, aluminum tolerance, phosphorus efficiency, flavonoid, carbohydrate

## Abstract

Aluminum (Al) toxicity and low phosphorus availability (LP) are the top two co-existing edaphic constraints limiting agriculture productivity in acid soils. Plants have evolved versatile mechanisms to cope with the two stresses alone or simultaneously. However, the specific and common molecular mechanisms, especially those involving flavonoids and carbohydrate metabolism, remain unclear. Laboratory studies were conducted on two wheat genotypes—Fielder (Al-tolerant and P-efficient) and Ardito (Al-sensitive and P-inefficient)—exposed to 50 μM Al and 2 μM Pi (LP) in hydroponic solutions. After 4 days of stress, wheat roots were analyzed using transcriptomics and targeted metabolomics techniques. In Fielder, a total of 2296 differentially expressed genes (DEGs) were identified under Al stress, with 1535 upregulated and 761 downregulated, and 3029 DEGs were identified under LP stress, with 1591 upregulated and 1438 downregulated. Similarly, 4404 DEGs were identified in Ardito under Al stress, with 3191 upregulated and 1213 downregulated, and 1430 DEGs were identified under LP stress, with 1176 upregulated and 254 downregulated. GO annotation analysis results showed that 4079 DEGs were annotated to the metabolic processes term. These DEGs were significantly enriched in the phenylpropanoid, flavonoid, flavone and flavonol biosynthesis, and carbohydrate metabolism pathways by performing the KEGG enrichment analysis. The targeted metabolome analysis detected 19 flavonoids and 15 carbohydrate components in Fielder and Ardito under Al and LP stresses. In Fielder, more responsive genes and metabolites were involved in flavonoid metabolism under LP than Al stress, whereas the opposite trend was observed in Ardito. In the carbohydrate metabolism pathway, the gene and metabolite expression levels were higher in Fielder than in Ardito. The combined transcriptome and metabolome analysis revealed differences in flavonoid- and carbohydrate-related genes and metabolites between Fielder and Ardito under Al and LP stresses, which may contribute to Fielder’s higher resistance to Al and LP. The results of this study lay a foundation for pyramiding genes and breeding multi-resistant varieties.

## 1. Introduction

Acid soils, defined as those with a pH lower than 5.5, pose numerous challenges to plant growth [1]. These soils are mainly distributed in the humid northern temperate zone and humid high-rainfall tropical areas, including Latin America, Africa, and Southeast Asia [2]. In China, acid soils are primarily located in the southern subtropical and tropical regions, which cover an area of 2.18 × 10^6^ km^2^ and account for approximately 22.7% of the country’s total land area [3]. Aluminum (Al) toxicity and inorganic phosphorus (Pi) deficiency are the two most considerable abiotic stresses in acid soil [4]. Al toxicity and Pi deficiency often occur simultaneously in acid soils, with phosphorus fixation in Al-P precipitates notably reducing phosphorus availability. Al toxicity impairs root development, diminishing plants’ ability to acquire Pi from the soil [5]. Al also directly interferes with P metabolism, such as glucose-6 phosphate, inorganic P (cytoplasm and vacuole) and ATP [6], as well as Pi signaling genes like *TaIPS1*, *TaSPX3*, and *TaSQD2* in wheat [7].

Consequently, crops grown in acid soils face the challenge of simultaneously increasing Pi uptake while minimizing the absorption of toxic Al^3+^. For instance, plants must balance Pi and Al absorption by altering root system morphology and architecture. Phosphorus deficiencies modify root growth, inhibiting primary root development while promoting lateral root formation [8]. Al toxicity impedes root cell expansion, elongation, and subsequent cell division. The binding of Al to cell walls reduces extensibility and elongation while increasing rigidity, thereby limiting Al^3+^ entry into root cells [5]. To enhance Pi acquisition, plants increase the contact area between roots and soil by growing more lateral roots and root hairs. This strategy is observed in both wild and cultivated plants, including Arabidopsis, maize, rice, wheat, and tomato [9,10].

Plants have developed defense mechanisms to simultaneously navigate the challenges of Al toxicity and LP in acid soils, encompassing external and internal forms [11]. Plants regulate Al uptake, translocation, and distribution to avoid Al toxicity [3] and efficiently take up Pi from the soil by orchestrating a set of transporters, which includes proton-coupled H_2_PO_4_^−^ symporter phosphate transporter (PHT) family proteins and SPX-domain phosphate transporters [12]. A well-documented external tolerance mechanism involves producing organic acids (OAs) from root apices, which is crucial for Al detoxification and Pi acquisition [1,13]. The malate transporter ALMT1 (aluminum-activated malate transporter 1) and its positive transcriptional regulator STOP1 (sensitive to proton rhizotoxicity 1) play an essential role in tolerance to Al toxicity [14] and Pi deficiency [15]. Two ABC transporters, STAR1/ALS3 (sensitive to Al rhizotoxicity 1/aluminum sensitive 3) mediate P deficiency-induced remodeling of root architecture [16,17]. The primary cell wall components pectin and hemicellulose can bind Al. The wall-associated kinases (WAKs) are involved in cell wall pectin metabolism-mediated responses to Al toxicity [18,19]. The internal mechanisms include isolating excess Al^3+^ into vacuoles and recycling P from vacuoles [13]. In addition, antioxidant enzymes, such as superoxide dismutase (SOD) and peroxidase (POD), are effective reactive oxygen species (ROS) scavengers that participate in Al and LP tolerance [20]. Plants activate common or specific physiological and metabolic reactions [5]. For example, Al toxicity and phosphorus deficiency significantly alter the accumulation of phenolic acids, organic acids, linoleic acid, starch, and sucrose in the roots of *P. massoniana*. Specifically, Al affects arginine and proline metabolism, while low phosphorus conditions impact phenylalanine metabolism [21]. Plant hormones (e.g., auxin, ethylene, strigolactones, and gibberellin) [8] and transcription factors (TFs) such as WRKY, MYB, bHLH, NAC, and ERF/AP2 [22] are key determinants of Al tolerance and phosphorus efficiency.

Like organic acids (OAs), flavonoids play a role in both internal and external Al detoxification by forming solid complexes with toxic Al ions [23]. For instance, alfalfa increased 7,4-dihydroxyflavone, dihydroquercetin, and formononetin accumulation in roots and secretion from root tips, leading to enhanced resistance to Al stress. The levels of superoxide anions (O_2_^−^) and malondialdehyde (MDA), as well as electrolyte leakage (EL), were significantly decreased when flavonoids were added under Al stress [24]. Al treatment also significantly increased the contents of dihydrotricin and silibinin, alleviating Al-induced oxidative stress in a tolerant lettuce genotype [25]. Flavonoids isolated from white lupin roots mobilized inorganic phosphorus and decreased soil microbial respiration, citrate mineralization, and soil phosphohydrolase activities but did not reduce soil ATP content [26]. Carbohydrates such as sucrose, glucose, fructose, and maltose are abundant in wheat and are essential as cell wall polysaccharides and signaling molecules [27]. Plants can provide glycolytically yielded energy (ATP) by upregulating genes involved in glycolysis, starch, and sucrose synthesis during periods of Al toxicity [28] and phosphorus (Pi) deficiency [29], thereby maintaining primary respiration.

Wheat (*Triticum aestivum* L., BBAADD, 2n = 6x = 42) is the most widely cultivated staple food crop. Aluminum (Al) toxicity and phosphorus (Pi) deficiency often coexist in acid soils, posing serious environmental challenges that affect and reduce wheat production worldwide [30]. Multi-omics techniques, such as transcriptomics, metabolomics, and proteomics, can be used to explore the molecular mechanisms of stress tolerance in wheat. For instance, transcriptome and metabolome analyses indicate that starch and sucrose metabolism, phenylpropane metabolism, and flavonoid metabolism play essential roles in wheat quality [31]. However, the specific and common molecular mechanisms of flavonoid and carbohydrate metabolism related to wheat tolerance to Al toxicity and phosphorus (Pi) deficiency remain unclear. Differentially expressed genes (DEGs) and differential metabolites (DMs) in two wheat cultivars—Fielder (Al-tolerant and Pi-efficient) and Ardito (Al-sensitive and Pi-inefficient)—were studied to identify the flavonoids and carbohydrates affected by Al toxicity and Pi deficiency. The results of the transcriptome and metabolome analyses were validated by quantitative PCR (qPCR) and measurements of flavonoid and carbohydrate contents. This study integrated transcriptome and metabolome analyses to reveal the specific and common mechanisms of flavonoid and carbohydrate metabolism in wheat under Al and Pi stresses, providing new insights into the molecular network of plant responses to multiple stresses in acid soils and identifying candidate genes for wheat Al and Pi tolerance, thus offering valuable genetic resources.

## 2. Results

### 2.1. Wheat Root Transcriptome Profiling in Response to Al and LP Stresses

Whole genome transcriptome sequencing analysis was applied to investigate the molecular responses of wheat roots under Al and LP stresses. A total of 18 cDNA libraries were constructed, combining two genotypes, three treatments, and three biological replicates. These libraries generated an average of 51.20 M clean reads. Quality control data indicated a Phred quality score Q30 ranging from 92% to 93.22%, with an average GC content of 54.39%. All samples exhibited an average total mapping rate of 91.41% and a unique mapping rate of 84.08%, demonstrating high-quality sequencing suitable for subsequent gene expression analysis. Finally, 118,095 genes (91,852 known and 26,243 new) and 192,506 transcripts (113,961 known and 78,545 new) were assembled. The functions of 91,852 genes and 113,961 transcripts were annotated with reference to KEGG, Swiss-Prot, Pfam, GO, COG, and NR databases (Table S2).

**Figure 1:**
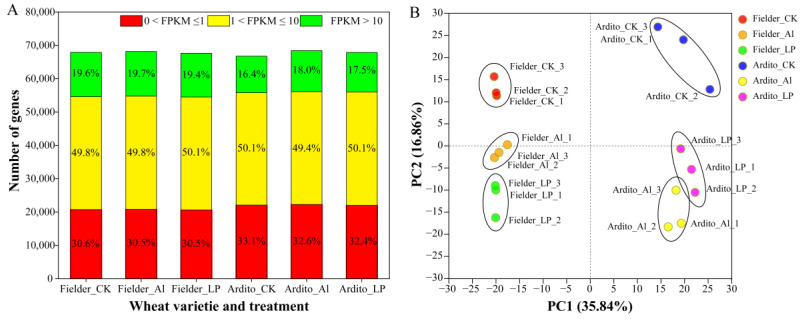
Gene expression profiling in each sample. (**A**) Numbers of detected genes in each sample. (**B**) Principal component analysis (PCA) of the RNA-Seq data from each sample.

In total, 67,902, 68,158, and 67,624 expressed genes were detected in the three treatments (CK, Al, and Pi) for Fielder, and 66,758, 68,430, and 67,864 for Ardito, respectively. The proportions of low-expressing (0 < FPKM ≤ 1), mid-expressing (1 < FPKM ≤ 10), and high-expressing (FPKM > 10) genes across the three treatments in each genotype were similar. Fielder exhibited a higher proportion of high-expressing genes compared to Ardito (Figure 1A). Principal component analysis (PCA) was conducted to identify the major sources of variation among the different treatments. The PCA results showed that samples within each group were reproducible, and noticeable differences existed between different varieties and treatment samples (Figure 1B). The correlation heatmap indicated that transcriptome activities within a genotype were more similar to each other than to identical treatments between genotypes (Figure S1).

### 2.2. Identified Differentially Expressed Gene Responses to Al and LP Stresses

**Figure 2:**
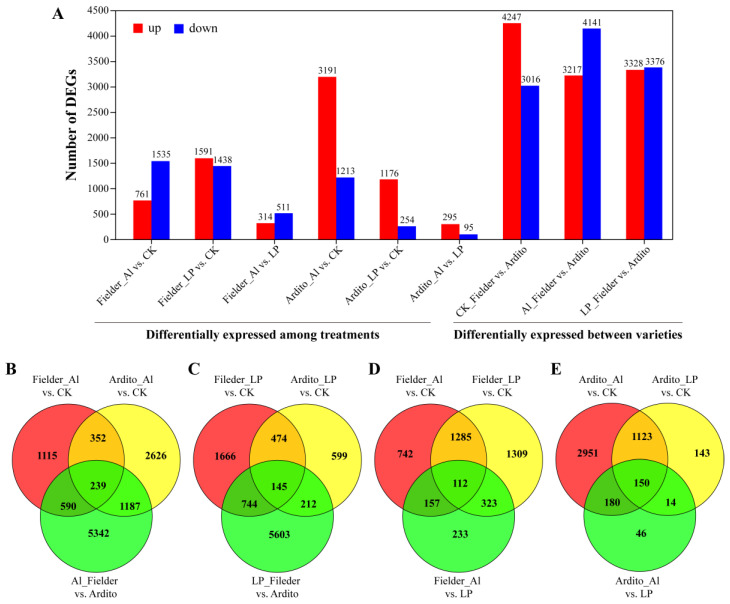
Al and LP responsive gene expression profiles. (**A**) The number of differentially expressed genes (DEGs) in each comparison. Fielder_Al vs. CK, Fielder_LP vs. CK, and Fielder_Al vs. LP represent the differentially expressed genes in the Fielder wheat variety under aluminum stress, low phosphorus stress, and control comparisons, respectively. Similarly, Ardito_Al vs. CK, Ardito_LP vs. CK, and Ardito_Al vs. LP represent the differentially expressed genes in Ardito wheat variety under aluminum stress, low phosphorus stress, and control comparisons, respectively. CK_Fielder vs. Ardito, Al_Fielder vs. Ardito, and LP_Fielder vs. Ardito represent the differentially expressed genes when comparing the two wheat varieties under control, aluminum toxicity, and low phosphorus stress conditions, respectively. (**B**–**E**) The Venn diagram displays the relationships between differentially expressed genes (DEGs) of various treatments (Al (**B**) and LP (**C**)) and varieties (Fielder (**D**) and Ardito (**E**)).

The differentially expressed genes (DEGs) were categorized into nine groups to compare their expression under Al and LP stresses in wheat roots (Figure 2A and Table S3). The comparison of the first six groups reflects the responses of genes under Al and LP stresses in the two wheat genotypes, and the last three groups reflect the changes in expression between genotypes. Based on the degree and importance of the observed stress impacts, six groups were selected for further evaluation. When subjected to Al and LP stresses in Fielder, the number of DEGs under LP stress (3029 DEGs, 1591 upregulated and 1438 downregulated) was higher than the number under Al stress (2296 DEGs, 1535 upregulated and 761 downregulated), while in Ardito, the number of DEGs under Al stress (4404 DEGs, 3191 upregulated and 1213 downregulated) was much higher than the number under LP stress (1430 DEGs, 1176 upregulated and 254 downregulated). A total of 7358 DEGs (3217 upregulated and 4141 downregulated) were expressed in Al_Fielder vs. Ardito and 6704 DEGs (3328 upregulated and 3376 downregulated) were expressed in LP_Fielder vs. Ardito. The number of DEGs was higher between varieties than between treatments (Figure 2A). The Venn diagram illustrates how the DEGs interacted with each other across various treatments and genotypes (Figure 2B and Table S3). Only 145 and 239 common DEGs under Al and LP stresses were identified in Fielder and Ardito, respectively. Moreover, 112 and 150 common DEGs among the three comparisons (Al vs. CK, LP vs. CK, and Al vs. LP) were identified in Fielder and Ardito, respectively (Figure 2B). These genes were considered to be valuable candidate genes contributing to common toxicity or tolerance mechanisms.

### 2.3. GO Annotations and KEGG Enrichment Analyses of DEGs under Al and LP Stresses

GO annotation and KEGG enrichment analyses were employed to examine the functions of these DEGs concerning the treatments and genotypes, and to compare the similarities and differences in metabolic pathways. DEGs were divided into 50 functional groups within the biological process (BP), cellular component (CC), and molecular function (MF), with the most prominent terms being the following (Table S4): binding (Go:0005488, 7124 genes) and catalytic activity (Go:0003824, 5595 genes) in BP; cell part (Go:0044464, 4332 genes) and membrane part (Go:0044425, 3742 genes) in CC; and cellular process (Go:0009987, 4260 genes) and metabolic process (Go:0008152, 4079 genes) in MF. These were the GO terms most commonly associated with the DEGs in all comparisons (Table S4). The responsive genes enriched in these GO terms differed between Fielder and Ardito under Al and LP. For instance, 622 and 766 DEGs under Al and LP stresses compared to CK in Fielder, and 1420 and 447 DEGs in Ardito, were annotated to the metabolic process term, respectively. In addition, 2035 and 1893 DEGs were annotated compared to Fielder and Ardito under Al and LP stresses (Table S4).

**Figure 3:**
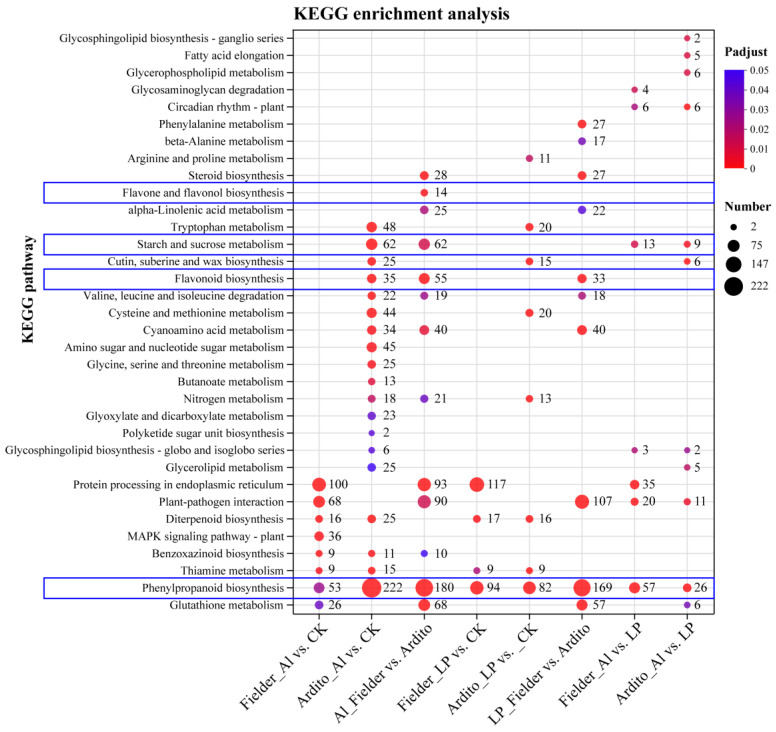
KEGG enrichment analysis for eight DEGs under Al and LP stresses. The bubble sizes indicate the numbers of genes involved in the terms and pathways. The content highlighted with blue frames represents the metabolic pathways related to flavonoids and carbon metabolism.

Consistent with the GO annotations, the KEGG enrichment analysis revealed the intricate metabolic pathways in wheat under Al and LP stresses (Figure 3). Some DEGs responsive to Al and LP stresses are enriched in common metabolic pathways. The DEGs of both Fielder and Ardito were significantly enriched in the diterpenoid biosynthesis (ko00904) and thiamine metabolism (ko00730) pathways under Al and LP stresses. However, some pathways responded specifically and explicitly to one genotype or treatment. For instance, several DEGs were significantly enriched in the benzoxazinoid biosynthesis (ko00402) pathway under Al stress only. Surprisingly, DEGs of all comparisons were significantly enriched in the phenylpropanoid biosynthesis (ko00940) pathway under Al and LP stresses, with 53 and 94 DEGs under Al and LP compared to CK in Fielder, and 94 and 222 DEGs in Ardito being enriched, respectively. A total of 180 and 169 DEGs were enriched compared to Fielder and Ardito under Al and LP stresses. In addition, several DEGs were enriched in the flavonoid biosynthesis (ko00941), flavone and flavonol biosynthesis (ko00943), and starch and sucrose metabolism (ko00500) pathways under Al and LP stresses in the two wheat genotypes. Those DEGs involved in phenylpropanoid and flavonoid biosynthesis and carbohydrate metabolism were studied to analyze the potential common and specific toxicity or tolerance mechanisms to Al toxicity and P deficiency.

### 2.4. DEGs Related to Transcription Factors

**Figure 4:**
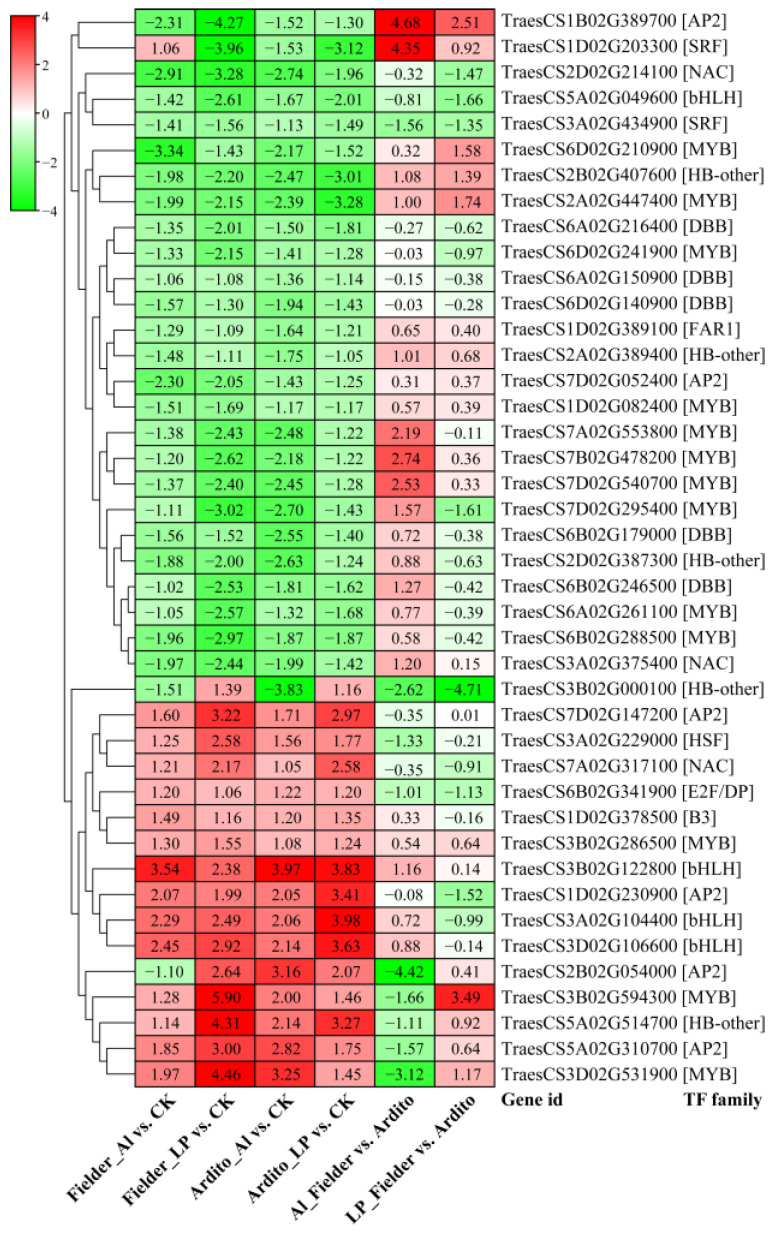
The changes in expression of common significantly differentially expressed TFs during Al and LP stresses in the two wheat genotypes.

When wheat roots were exposed to Al and LP stress, a significant number of transcription factors (TFs) displayed varying levels of expression. A total of 652 TFs were identified, spanning 29 TF families. The top five most abundant TF families were *MYB* (136), *AP2* (84), *WRKY* (58), *bHLH* (56), and *NAC* (47) (Table S5). Among these TFs, 42 were significantly differentially expressed under Al and LP stresses in the two wheat genotypes, encompassing families such as *AP2*, *B3*, *bHLH*, *DBB*, *E2F/DP*, *FAR1*, *HB-other*, *HSF*, *MYB*, *NAC*, and *SRF*. The majority of TFs exhibited a consistent expression trend, with three *AP2*s, three *bHLH*s, three *MYB*s, one *B3*, one *ANC*, one *HSF*, and one *HB-other* being upregulated under Al and LP stresses. In contrast, 10 *MYB*s, five *DBB*s, three *HB-others*, two *AP2*s, two *NAC*s, one *SRF*, one *bHLH*, and one *FAR1* were downregulated in the two wheat genotypes. Three TFs showed a different expression pattern: one *HB-other* (TraesCS3B02G000100) was downregulated under Al but upregulated under LP, one *AP2* (TraesCS2B02G054000) was downregulated in Fielder under Al but upregulated under LP, while one *SRF* (TraesCS1D02G203300) exhibited an opposite trend (Figure 4).

### 2.5. Confirmation of RNA-Seq Data Using qRT-PCR

**Figure 5:**
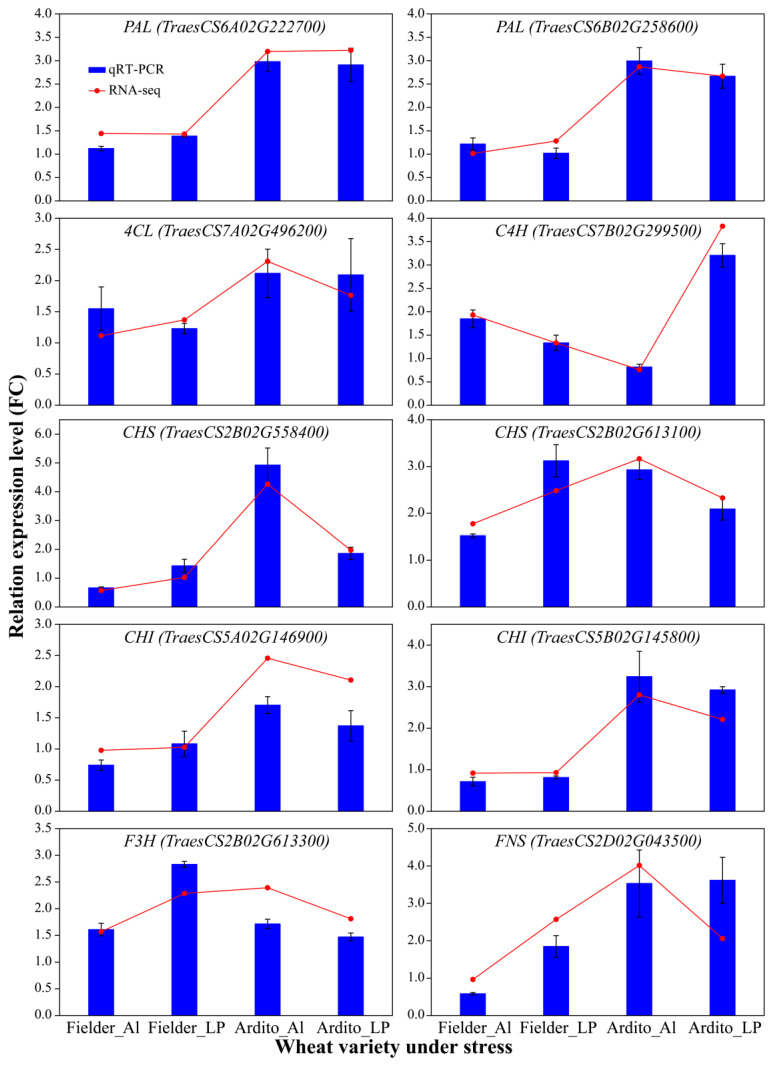
The relative expression levels detected by qRT-PCR using the 2^−ΔΔCT^ method are shown by the blue bars, while the red line graph represents the FPKM ratio, indicating transcript abundance in the RNA-Seq data. *TaActin* was used as an internal control. Values are means ± SD; *n* = 4. PAL—phenylalanine ammonia-lyase; 4CL—4-coumarate-CoA ligase; C4H—cinnamic acid 4-hydroxylase; CHS—chalcone synthase; CHI—chalcone isomerase; F3H—flavanone 3-hydroxylase; FNSI—flavone synthase.

To assess the quality of the RNA-Seq data and confirm the DEGs, the expression levels of 10 genes related to the phenylpropanoid and flavonoid biosynthesis pathways were measured using qRT-PCR. Correlation analysis with the transcriptome data showed that the qRT-PCR results supported the RNA-Seq quantification results (R = 0.8885) (Figure S2). The expression levels of genes encoding 4CL, C4H, CHS, and F3H were upregulated, while one gene encoding CHI was downregulated under Al and LP stresses in Fielder. The expression levels of *CHS*, *CH*I, and *FNS* were upregulated under LP but downregulated under Al in Fielder. The expression levels of genes encoding PAL, C4H, 4CL, CHI, and FNS were significantly upregulated under Al and LP stresses in Ardito. The gene encoding C4H was downregulated under Al stress but upregulated under LP stress (Figure 5). These genes can be used to further verify the reliability of the transcriptional data.

### 2.6. Joint Analysis of Genes and Metabolites Involved in Flavonoid Metabolism

**Figure 6:**
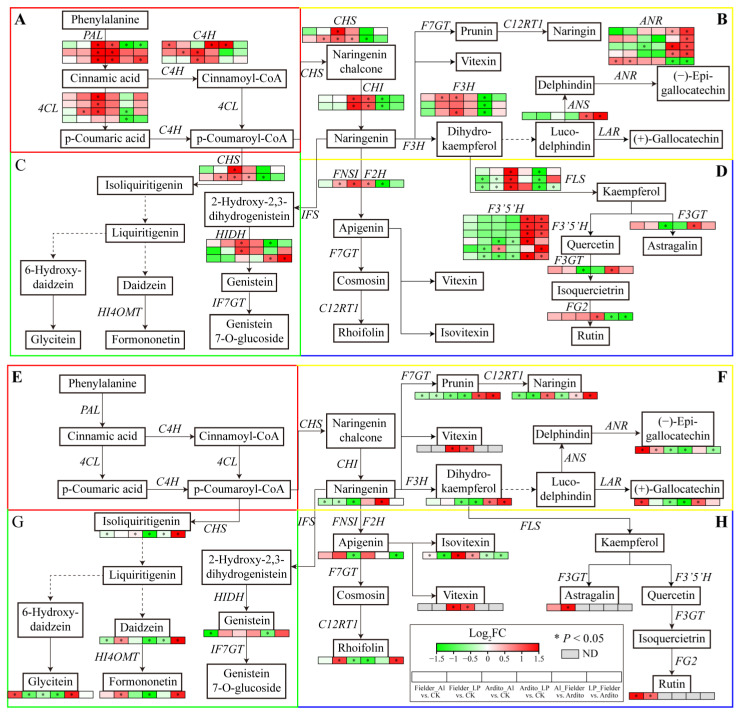
Joint analysis of flavonoid metabolism genes and metabolites. The changes in expression of DEGs (**A**–**D**) and metabolites (**E**–**H**) related to the pathways of (**A**,**E**) general phenylpropanoid, (**B**,**F**) flavonoid, (**C**,**G**) isoflavonoid, and (**D**,**H**) flavone and flavonol biosynthesis under Al and LP stresses in the two wheat genotypes. ND—not detected. ANS—anthocyanidin synthase, ANR—anthocyanidin reductase; F3′5′H—flavonoid 3′,5′-hydroxylase; F3GT—flavonol 3-O-glucosyltransferase; FG2—anthocyanidin-3-O-glucoside rhamnosyltransferase; HIDH—2-hydroxyisoflavanone dehydratase.

The KEGG enrichment analysis showed that several DEGs were significantly enriched in the phenylpropanoid, flavonoid, and flavone and flavonol biosynthesis pathways (Figure 3). The genes involved in flavonoid biosynthesis differed between Al and LP stresses in the two wheat genotypes. Some genes were significantly upregulated under LP stress in Fielder, including *4CL*, *C4H*, *CHS*, *F3H*, and *FNSI*, but only one *C4H* was upregulated under Al stress. Al and LP stresses upregulated *ANR* expression and downregulated *FLS* expression in Fielder. Similarly, the genes encoding 4CL, C4H, CHS, HIDH, F3H, and FLS were significantly upregulated under Al stress in Ardito, but only one *FG2* gene was upregulated under LP stress. Al and LP stresses upregulated *PAL*, *CHI*, and *CHS* expression and downregulated *F3′5′H* expression in Ardito. In addition, four *F3′5′H*, three *ANR*, one *ANS*, and one *HIDH* gene were upregulated, and one *FLS* and one *FG2* gene were downregulated compared to Fielder and Ardito under Al and LP stresses (Figure 6 and Table S7).

Flavonoid metabolism-targeted metabolomics analysis was conducted to further investigate the flavonoid synthesis mechanism in wheat roots within 19 differential flavonoid metabolites that significantly influenced two wheat roots. As with the transcriptome results, the flavonoid metabolites showed different responses under Al and LP stresses in the two wheat genotypes. The daidzein, formononetin, rhoifolin, and astragalin contents were significantly increased under LP stress, and the (+)-gallocatechin and glycitein contents were increased under Al stress in Fielder. Al and LP stresses increased the (−)-epigallocatechin and rutin contents and decreased the prunin, naringenin, and naringin contents. The naringin and isoliquiritigenin contents were increased under Al stress but decreased under LP stress. The daidzein and formononetin contents were decreased under LP stress but not significantly under Al stress, and the apigenin and naringenin contents showed opposite trends. Al and LP stresses increased the vitexin and isovitexin contents and decreased the glycitein, prunin, dihydrokaempferol, (−)-epigallocatechin, and (+)-gallocatechin contents in Ardito. It was found that the contents of the eight and nine differential flavonoid metabolites were increased in Fielder compared to Ardito under Al and LP stresses, respectively. The prunin, naringin, and dihydrokaempferol contents were increased compared to Fielder in Ardito under Al and LP stresses. In addition, the rutin was detected in Fielder, and the vitexin was detected in Ardito only (Figure 6 and Table S8).

The changes in the expression levels of these genes were consistent with the changes in the contents of related metabolites regulated by the enzymes encoded by the genes, which can be used to verify the reliability of transcriptional data. For instance, the genes encoding PAL, 4CL, C4H, CHI, CHS, and FNSI related to the general phenylpropanoid and flavonoid biosynthesis pathways were significantly upregulated when the vitexin and isovitexin contents increased in Ardito, but not significantly in Fielder. The astragalin and rutin contents significantly increased in Fielder but were not detected in Ardito, which is consistent with the elevated expressions of the upstream genes encoding F3GT, FG2, and F3′5′H, which were upregulated in Fielder but downregulated in Ardito, although not significantly. One ANR gene was significantly upregulated in Fielder, while another was downregulated in Ardito, so the (−)-epigallocatechin and (+)-gallocatechin contents showed the same trends in the two wheat genotypes (Figure 6).

### 2.7. Joint Analysis of Carbohydrate Metabolism Genes and Metabolites

The transcriptome data showed that several DEGs were enriched in starch and sucrose metabolism, glycolysis/gluconeogenesis, and the TCA cycle pathway. Most genes involved in carbohydrate metabolism were downregulated under Al and LP stresses. One *INV* under Al and LP stresses, one *SUS*, *TPP*, and *ALDO* under Al stress, and one *INV* and two *SUS*s under LP stress were significantly downregulated in Fielder. One *PPDK* under Al, two *SUS*s and one *TPP* under LP stress were upregulated. One *INV*, one *SUS*, two *PFP*, and one *PGK* under Al stress, and one *TPS* under LP stress were downregulated. Al and LP stresses upregulated one *SUS* and two *TPP*s and downregulated the expression of one *TPS* in Ardito. In addition, the genes encoding SUS, ALDO, ACO, and IDH were upregulated and the genes encoding TPP, ALDO, GAPDH, OGK, MDH, and FUM were downregulated compared to Fielder and Ardito.

Carbohydrate metabolism-targeted metabolomics analysis was conducted further to investigate the carbohydrate synthesis mechanism in wheat roots. A total of 15 differential carbohydrate metabolites were identified (Tables S7 and S9). Consistent with the transcriptome results, most carbohydrate metabolites were downregulated under Al and LP stress compared to CK but showed an upregulation compared to Fielder and Ardito. For instance, the glucose, glucose-6-phosphate, fructose, fructose-6-phosphate, trehalose-6-phosphate, pyruvate, citrate, cis-aconitate, isocitrate, and succinate contents were higher in Fielder than in Ardito. A few metabolites were upregulated within the trehalose-6-phosphate content under Al and LP stress, the fructose-6-P content under Al stress, and the citrate and fumarate contents under LP stress in Fielder. The glyceric acid contents under Al and LP stress, cis-aconitate contents under Al stress, and glyceraldehyde contents under LP stress were increased in Ardito.

**Figure 7:**
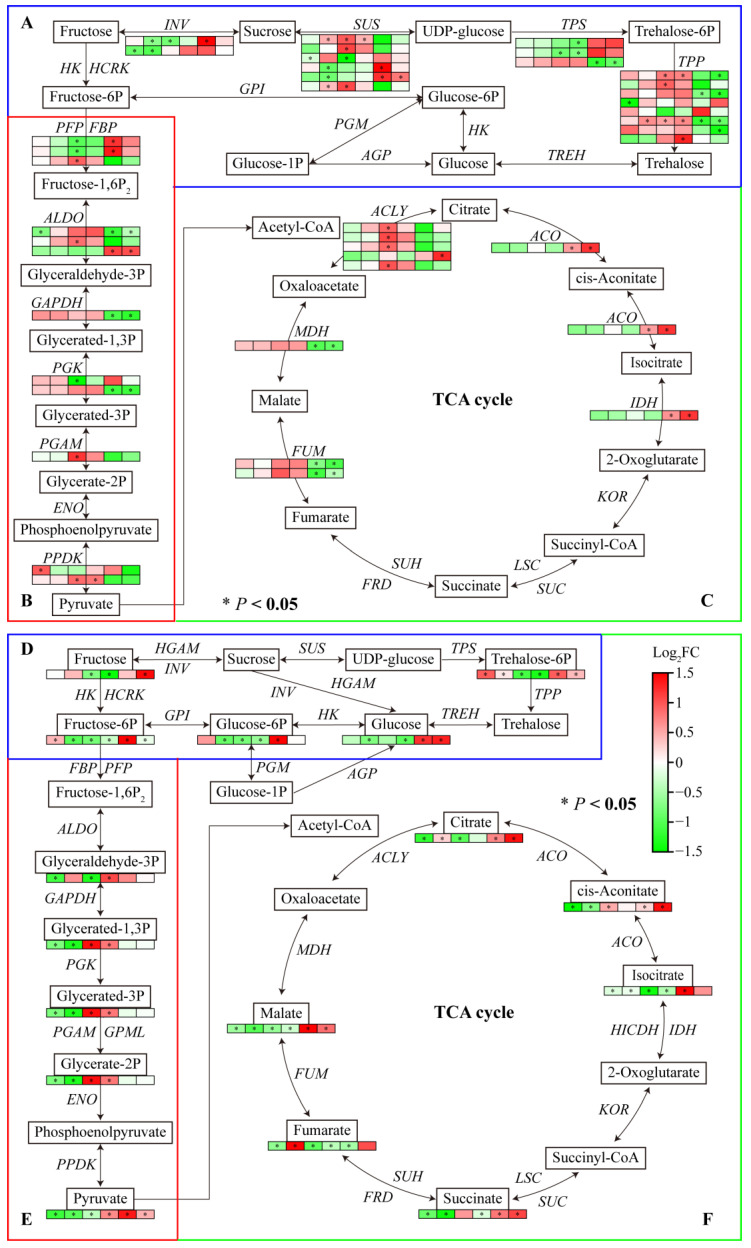
Joint analysis of carbohydrate metabolism genes and metabolites. The changes in expression of DEGs (**A**–**C**) and metabolites (**D**–**F**) related to the pathways of (**A**,**D**) starch and sucrose metabolism, (**B**,**E**) glycolysis/gluconeogenesis, and (**C**,**F**) TCA cycle under Al and LP stresses in the two wheat genotypes. TPP—trehalose-phosphatase; TPS—alpha, alpha-trehalose-phosphate synthase; SUS—sucrose synthase; INV—β-fructofuranosidase; ALDO—fructose-bisphosphate aldolase; GAPDH—glyceraldehyde-3-phosphate dehydrogenase; PGK—phosphoglycerate kinase; PGAM—phosphoglycerate mutase; PFP—phosphofructokinase; FBP—fructose-1-6-bisphosphatase; PPDK—pyruvate, phosphate dikinase; ACLY—ATP-citrate synthase; IDH—isocitrate dehydrogenase; ACO—aconitate hydratase; MDH—malate dehydrogenase; SDH—succinate dehydrogenase; FUM—fumarate hydratase.

Most of the changes in the expression levels of these genes were inconsistent with the changes in the contents of related metabolites regulated by the enzymes encoded by the genes (Figure 7). For instance, the genes encoding INV, SUS, PFP, ALDO, ACLY, ACO, and IDH were significantly upregulated, and the glucose, glucose-6-phosphate, fructose, fructose-6-phosphate, trehalose-6-phosphate, pyruvate, citrate, cis-aconitate, isocitrate, and succinate contents were higher in Fielder than Ardito. Two *TPS* genes were downregulated, and the trehalose-6-phosphate content significantly decreased in Ardito. One *ALDO* gene and one *PGAM* gene were upregulated in Ardito and one *ALDO* gene was downregulated in Fielder, which caused the glyceric acid content to decrease in Fielder and increase in Ardito.

## 3. Discussion

Al toxicity and LP dramatically inhibit crop growth and development in acid soils. Breeding cultivars for Al tolerance and P efficiency, and understanding the molecular mechanisms involved in adaptation to Al and LP stresses, are key components of sustainable agriculture [13]. However, few studies have simultaneously investigated the transcriptome and metabolome or integrated both transcriptome and metabolome analyses. To the best of our knowledge, this is the first study to report on transcriptomic and metabolomic responses to Al and LP stresses, providing new insights into the adaptive strategies of wheat for further research.

The KEGG enrichment analysis showed that several DEGs were enriched in the phenylpropanoid, flavonoid, flavone and flavonol biosynthesis pathways under Al and LP stresses in the two wheat genotypes (Figure 3). Our study reveals significant alterations in the expression of genes involved in flavonoid biosynthesis, underscoring the pivotal role of flavonoids in wheat tolerance to Al and LP. Recent integration of metabolomics and transcriptomics has highlighted that wheat’s response to LP stress involves flavonoid metabolism [32]. The metabolomics analysis showed that wheat with high resistance to Al toxicity has higher levels of flavonoid metabolites [33]. These results suggest that genes involved in flavonoid synthesis are crucial for wheat tolerance to Al and LP stresses. However, the response of flavonoid metabolism-related genes such as *4CL*, *CHS*, *F3H*, *FLS*, and *FNSI* to Al and LP stresses differs between the two wheat genotypes (Figure 6). The first three steps in the phenylpropanoid pathway catalyzed by PAL, 4CL, and CHS are known as the general phenylpropanoid pathway. *PAL*, *C4H*, and *4CL* are often coordinately expressed and correlated with anthocyanin and flavonol content in response to stress [34]. Dihydroflavonol catalysis of F3H is a crucial intermediate metabolite and a critical branch point in the flavonoid biosynthesis pathway. The dihydroflavonols are converted to the flavonols kaempferol, quercetin, and myricetin by FLS [35]. Flavones are produced from flavanones by flavone synthase (FNS); for instance, naringenin, liquiritigenin, eriodictyol, and pentahydroxyflavanone can be converted to apigenin, dihydroxyflavone, luteolin, and tricetin, respectively [35]. The targeted metabolome results corroborate the transcriptome data, showing that 19 differential flavonoid metabolites significantly influenced the two wheat roots (Table S8). Many flavonoids are reported in plant resistance to Al stress by forming Al-chelating complexes or scavenging free radicals through their Al-binding affinity [36]. For instance, catechin and rutin are found in the root exudates of *Rumex acetosa*, an aluminum resistant plant [23]. The contents of (−)-epigallocatechin and rutin were increased under Al and LP in Fielder but not in Ardito, indicating Fielder’s ability to respond to Al stress through upregulation of gene expression and elevation of (−)-epigallocatechin and rutin contents. Naringin and isoliquiritigenin, which are involved in Al tolerance [36], increased under Al stress but not significantly under LP stress in Ardito. Many of the highly accumulated flavonoid compounds identified in Fielder under LP stress but not under Al stress have not previously been reported in the literature as functioning in resistance to LP stress, including (+)-gallocatechin, astragalin, rhoifolin, daidzein, and formononetin. Flavonoids facilitate P solubilization and cooperate with beneficial microorganisms in the rhizosphere, contributing to P acquisition and utilization [26,37]. These flavonoids may help Fielder to tolerate LP.

Due to their inability to balance the absorption of Al and P by altering their root structures, plants have developed internal and external mechanisms to resist Al toxicity and P deficiency [13]. Similarly to organic acids, flavonoids participate in both internal and external Al detoxification by forming solid complexes with toxic Al ions [24,38]. The accumulation of flavonoids in roots can alter root structure (internal mechanism) and be released to dissolve soil P (external mechanism), thereby obtaining more Pi [39,40]. Transcriptome and metabolomics data consistently indicate that root flavonoid metabolism significantly impacts both wheat genotypes, but with differences between Al and LP stresses. The expression of flavonoid-related genes and the levels of flavonoid metabolites in Fielder are more significantly affected by LP stress than by Al stress, suggesting that the increased synthesis of flavonoids in the roots primarily contributes to their secretion outside the wheat roots (external mechanism). This facilitates Pi acquisition and can also bind to Al outside the roots to prevent it from entering root cells. In Ardito, the increased synthesis of flavonoids in the roots may play a more critical role within the roots (internal mechanism). This is because flavonoids are more actively involved in binding with Al entering the cells in response to Al stress, thereby reducing P absorption. These results suggest that the differences in flavonoid responses to Al and LP stresses contribute to the varying tolerance levels between the two wheat genotypes. Additionally, the content of some flavonoids, including (−)-epigallocatechin, rutin, vitexin, and isovitexin, increases under both Al and LP stress in the two wheat genotypes, playing a joint role in clearing ROS caused by Al and LP stress [13,41].

Carbohydrate metabolism comprises sugar, glycolysis, TCA cycle, and organic acid metabolism. The transcriptome data showed that several DEGs were enriched in starch and sucrose metabolism, glycolysis/gluconeogenesis, and TCA cycle pathways (Figure 4), suggesting that Al and LP affect the metabolic product content of carbohydrate metabolism in wheat roots. The relative concentrations of metabolites such as 6-fructose phosphate, 6-glucose phosphate, inositol phosphate, and glycerol 3-phosphate were dramatically reduced in Pi-deficient maize leaves and roots [42]. Al toxicity reduced ATP production and accumulation in rice roots, and nonstructural carbohydrates can directly provide energy for plant growth [43]. Similarly, Al and LP stresses reduced the carbohydrate genes and metabolites in two wheat genotypes. However, most genes related to carbohydrate metabolism were significantly upregulated compared to Fielder and Ardito under Al and LP stresses. Consistent with the transcriptome results, carbohydrate-targeted metabolomics analysis showed that glucose, glucose-6-phosphate, fructose, fructose-6-phosphate, and trehalose-6-phosphate were significantly higher in Fielder compared to Ardito (Figure 7). The P-efficient genotype had a remarkable ability to maintain phosphorylated sugars (i.e., glucose-6-phosphate and fructose-6-phosphate) by upregulating genes involved in glycolysis, starch, and sucrose synthesis, which are important for glycolysis as well as the biosynthesis of sugars and starch in wheat [33]. Similarly, high carbohydrate contents and gene expression have been reported in rice [44] and cotton [45]. The Al-tolerant rice cultivar [28] and citrus species [46] could produce more glycolytically yielded energy (ATP) by enhancing the glycolytic pathway in Al-stressed roots. Plants produce ATP and CO_2_ through photosynthetic product breakdown (e.g., glucose) by respiration to promote root growth and development or maintain root activity for nutrient and water uptake and translocation. Sucrose, the end product of photosynthesis, is responsible for energy metabolism and the synthesis of complex carbohydrates. ATP in plant cells is mainly generated by the TCA cycle in the mitochondria [27,45]. Pi deficiency increases carbohydrate translocation to the roots via the phloem to favor root growth for better acquisition of Pi from soil [47]. The differences in carbohydrate metabolite accumulation between genotypes in the roots suggest that Fielder better maintains the phosphorylated sugars required for sugar and starch biosynthesis, thereby regulating the metabolic processes needed for energy and carbon skeleton production to support root growth.

Plants utilize organic acid anions (OAs), primarily malate, citrate, and oxalate, to combat Al toxicity and phosphorus (P) deficiency. OAs secreted by roots into the rhizosphere can chelate Al^3+^ externally and mobilize phosphate (Pi). Concurrently, endogenous OAs can sequester Al^3+^ into the vacuole and release free Pi for metabolic processes [48]. Plants are capable of synthesizing malate and citrate, which are involved in the TCA cycle [48]. ATP-citrate lyase (ACLY) catalyzes the transformation among acetyl-CoA, oxaloacetate, and citrate, a critical step in pyruvate metabolism entering the TCA cycle, particularly under low phosphorus conditions. In this study, one ACLY showed higher expression in Fielder roots compared to Ardito, suggesting its crucial role in promoting the TCA cycle. Additionally, citrate, cis-aconitate, isocitrate, and succinate contents, as well as the expression of IDH and ACO, were upregulated in Fielder compared to Ardito. However, malate content and MDH expression were downregulated. Wheat roots release OAs (mainly malate) to form Al-OA complexes in the rhizosphere, mitigating Al toxicity [49]. Citrate efflux occurs constitutively in Brazilian wheat cultivars Carazinho, Maringa, Toropi, and Trintecinco [50]. Under Al stress, wheat secretes malate as a general mechanism to reduce Al^3+^ absorption. In non-Brazilian varieties, such as Fielder and Ardito, other organic acids, including citrate, cis-aconitate, isocitrate, and succinate, synthesized from the TCA cycle, may help to chelate aluminum ions and reduce Al toxicity within cells.

Transcription factor proteins (TFs) play crucial roles in regulatory and signaling networks to respond to Al and LP conditions [13]. AtSTOP1 and OsART1 are two related members of the Cys_2_His_2_-type zinc finger (C_2_H_2_) protein family TFs that confer Al resistance functions in *Arabidopsis* and rice [51]. Phosphate starvation response (PHR) transcription factors, such as AtPHR1 in *Arabidopsis* and OsPHR2 in rice, act as central regulators of systemic Pi signaling. AtPHR1/OsPHR2 activates a large set of low Pi-responsive genes by binding to PHR1 binding site (P1BS) elements [5]. TFs may offer the best option for pleiotropic control of multiple abiotic stress genes due to their small and often multiple binding sequences in the genome. TFs such as C_2_H_2_, MYB, WRKY, ERFs, NAC, and bHLH may be critical determinants for a plant’s ability to tolerate Al toxicity and P deficiency and withstand drought conditions in acid soil [22]. Promising connections may also exist between plant adaptation to Al toxicity and P deficiency through the regulation of TFs [22]. Our transcriptome results showed that 42 TFs, including MYB, bHLH, NAC, and AP2/ERF, were differentially expressed simultaneously under Al and LP stresses. Importantly, their expression changes after Al and LP treatments were similar, indicating that these TFs are associated with response to Al toxicity and P deficiency. Several TFs have been identified as hub genes involved in flavonoid, carbohydrate, and P metabolism [45]. MsMYB741 transcriptionally activates *MsPAL1* and *MsCHI* expression to increase flavonoid accumulation in roots and secretion from root tips, leading to increased resistance of alfalfa to Al stress [24]. MhMYB15 actively responds to *Bacillus B2*, regulating the accumulation of flavonoids and phosphorus uptake, thereby influencing plant growth and development [52]. MYB-related transcription factor PHR1 is involved in carbohydrate metabolism. The knockout mutant has an altered phosphate (Pi) allocation between root and shoot and accumulates less anthocyanins, sugars, and starch than P-starved WT [53]. Thirteen common MYB TFs were identified, suggesting that MYB TFs may be involved in flavonoid and carbohydrate metabolism. Further investigation into the specific functions and mechanisms of key flavonoid metabolism genes and transcription factors is needed. Understanding their roles in Al and Pi tolerance will aid in the precise breeding of resistant wheat varieties.

Al toxicity and phosphorus (Pi) deficiency pose serious environmental challenges that impact and reduce wheat production worldwide [30]. Previous studies have consistently reported a positive correlation between phosphorus efficiency and Al tolerance in wheat [7]. Al-tolerant wheat plants exhibit high P contents or efficient P acquisition and translocation to shoots [54]. Given the coexistence of Al toxicity and P deficiency in acid soils, researchers have been motivated to study the interaction between these two stress factors and plant adaptations to acid soils [13]. Our results indicate that Fielder demonstrates better tolerance to Al and LP stress compared to Ardito. In Fielder, genes related to flavonoid and carbohydrate metabolism, as well as transcription factors (TFs) such as MYB, bHLH, NAC, and AP2/ERF families, were activated to manage Al and LP stresses. These identified genes and pathways can be targeted for gene pyramiding and the breeding of multi-tolerant varieties. Fielder is an easily transformable wheat variety and serves as a preferred material for gene function research through techniques such as overexpression and gene editing [55]. This greatly facilitates subsequent functional validation and utilization of genes related to aluminum tolerance and phosphorus efficiency. Future research should leverage these insights for gene pyramiding and molecular breeding to develop wheat varieties with enhanced multi-tolerance.

## 4. Materials and Methods

### 4.1. Plant Materials and Treatment

Based on the morphological performance in preliminary experiments, two wheat genotypes—Fielder (Al-tolerant and P-efficient) and Ardito (Al-sensitive and P-inefficient)—were used in this study. Healthy seeds were surface sterilized in a solution of 0.5% NaClO (*v*/*v*) for 30 min and then rinsed thoroughly with deionized water. These sterilized seeds were then placed on wet paper in a petri dish for 4 days at 4 °C for germination. Uniform seedlings were selected and transferred to a 10 L barrel with continuously aerated 1/5 Hoagland nutrient solution (pH 4.2) for a 2-day acclimation to low pH. Subsequently, the seedlings were subjected to stress by replacing fresh 1/5 Hoagland nutrient solution with an addition of 50 µM KAl(SO_4_)_2_ solution (for Al stress), a reduced NaH_2_PO_4_ solution (2 µM, for LP stress), and normal Pi levels (200 µM, for control). The growth chamber environments were 14/10 h of light/dark period, 60–80% relative humidity, 23 °C, and 400 μmol m^−2^·s^−1^ illumination level. Each treatment was replicated three times (3 barrels) and the pH of the solution was maintained at 4.2 by adding 0.5 mM HCl. After 4 d of treatments, approximately 1 cm length wheat root apices were harvested separately in triple biological repeats, immersed immediately in liquid nitrogen, and stored at −80 °C until transcriptomics and metabolomics analyses were conducted. All chemicals used were of analytical grade and were purchased from Sigma-Aldrich (St. Louis, MO, USA).

### 4.2. RNA Extraction, Library Preparation, Sequencing, and Read Mapping

The total RNA was separately extracted from the collected 1 cm root apices using TRIzol reagent (Invitrogen Co., Carlsbad, CA, USA) following the manufacturer’s instructions. Subsequently, the Plant RNA Purification Reagent (Invitrogen) was employed to purify the RNA samples. The quality, quantification, and integrity were determined using a 5300 Bioanalyzer (Agilent Co., Santa Clara, CA, USA) and ND-2000 (Thermo Fisher NanoDrop, Waltham, MA, USA), respectively. RNA samples meeting integrity number values above 7.5 and total amounts of ≥1 µg, and 28S: 18S ≥ 1.0 were utilized to construct the transcriptome libraries for sequencing, following the manufacturer’s protocols (Illumina, San Diego, CA, USA) at Majorbio Bio-pharm Biotechnology Co., Ltd. (Shanghai, China). In brief, mRNA was isolated from the total RNA by poly(A) selection using oligo (dT) beads, followed by fragmentation into short fragments. Subsequently, cDNA was synthesized using a Superscript double-stranded cDNA synthesis kit (Invitrogen) with random hexamer primers. The synthesized cDNA was subjected to library construction according to Illumina’s library construction protocol. The cDNA target fragments of 300 bp were PCR amplified using Phusion DNA polymerase (NEB, Ipswich, MA, USA). After quantification with Qubit 4.0, the paired-end RNA-seq library was sequenced using the NovaSeq 6000 sequencer with a 2 × 150 bp read length.

Finally, 18 RNA-seq transcriptome libraries (2 genotypes × 3 treatments × 3 biological replicates) were constructed and sequenced. The raw paired-end reads were trimmed and quality controlled using FASTP [56] with default parameters, resulting in high-quality sequencing data (clean data). The clean reads were then separately aligned to the wheat genome using the orientation mode on HISAT2 software Ver. 2.2.1 [57]. The mapped reads of each sample were assembled by StringTie Ver. 1.3.6 [58] in a reference-based approach for subsequent transcript assembly and expression calculation. The assembled genes were annotated against the public databases of NCBI non-redundant protein sequences (NRs) the Protein family (Pfam), Clusters of Orthologous Groups of Proteins (COG), Swiss-Prot, Kyoto Encyclopedia of Genes and Genomes (KEGG), and Gene Ontology (GO) libraries. The RNA-seq data were uploaded to the Comprehensive Gene Expression Database under the accession number PRJNA1033153.

### 4.3. Differential Expression and Functional Enrichment Analysis

The expression levels of each transcript were calculated using the Fragments Per Kilobase per Million reads (FPKM) method to identify differential expression genes (DEGs) between samples. The gene abundances were quantified using RSEM software Ver. 1.3.3 [59]. DESeq2 was used to identify DEGs based on the criteria of |log_2_FC| ≥ 1 and FDR ≤ 0.05 [60]. Cluster heat maps were generated using Toolkit for Biologists (TB) tools Ver. 2.042 with default settings [61]. The Plant TFDB database was used for predicting and analyzing transcription factors (TFs), while TFs were identified by BLAST and then subjected to functional annotation and enrichment analysis. The GO annotation and functional classification of DEGs were performed using Blast2GO based on the GO database [62]. KEGG enrichment pathways were determined using KOBAS 2.0 at Bonferroni-corrected *p*-values ≤ 0.05, which were compared with the whole transcriptome background [63].

### 4.4. Quantitative Real-Time PCR (qRT-PCR) Validation

Ten differentially expressed genes involved in phenylpropanoid and flavonoid biosynthetic pathways were selected from the RNA-seq data to validate the transcriptome results. The identical RNA/cDNAs for RNA-seq were used as templates to determine their transcript levels via the qRT-PCR method. The first-strand cDNA was synthesized using the PrimeScript™ RT Master Mix (Takara Bio Inc., Osaka, Japan). The gene-specific primers were designed by Primer3 web v4.1.0 (https://primer3.ut.ee/ (accessed on 23 October 2023)) and listed in Table S1. A 10 μL PCR reaction mixture contained TB Green^®^ Premix Ex Taq™ II (Takara Standard Co., Ltd., Osaka, Japan)—5 μL; 10 μM primers—each 0.4 μL; cDNA template—1 μL; and ddH_2_O—3.2 μL. The mixture was amplified using the LightCycler 480 machine (Roche, Basel, Switzerland) with a two-step thermal cycling method (95 °C for 30 s, followed by 40 cycles of 95 °C for 5 s and 60 °C for 30 s). TaActin (accession number XM_044554036.1) served as an internal control for calculating relative gene transcript levels using the 2^−ΔΔCT^ method [64]. Four technical replicates were performed for qRT-PCR and representative results from at least three biological replicates are shown for each gene.

### 4.5. Targeted Metabolite Extraction and Profiling

The frozen wheat root apices were dispatched to Shanghai Majorbio Biopharmaceutical Biotechnology Co., Ltd. (Shanghai, China) for targeted metabolite extraction and analysis. Precision weighing was conducted on 0.1 g root tip samples and metabolite extraction was performed in a low-temperature environment. The supernatant was filtered through a 0.2 μm membrane and then transferred to the injection vial for analysis. Flavonoid and carbohydrate standard solutions were prepared with concentration gradients. LC-ESI-MS/MS (UHPLC-Qtrap) was used to qualitatively and quantitatively detect the target substances in the samples. The chromatographic apparatus was the ExionLC^TM^ CAD system (AB Sciex, Los Angeles, CA, USA). The mass spectrometry system was the SCIEX QTRAP 6500+ (AB Sciex, Los Angeles, CA, USA).

The specific conditions and parameters for flavonoid determination were as follows: Agilent Poroshell 120EC-C18 (3 × 100 mm, 1.9 μm, Agilent Co., Santa Clara, CA, USA) liquid chromatography column; column temperature—40 °C; injection volume—1 μL; mobile phase A—0.1% formic acid in water; mobile phase B—0.1% formic acid in methanol; negative mode detection; curtain gas—35 psi; collision gas—medium; IonSpray Voltage—−4500 V; temperature—350 °C; Ion Source Gas1—55 psi; Ion Source Gas2—55 psi.

The specific conditions and parameters for carbohydrate determination were as follows: Waters HSS T3 (2.1 × 150 mm, 1.8 μm, Waters, Milford, MA, USA) liquid chromatography column; column temperature—40 °C; injection volume—2 μL; mobile phase A—0.03% formic acid in water; mobile phase B—0.03% formic acid in methanol; positive/negative mode detection; curtain gas—35 psi; collision gas—medium; IonSpray Voltage—+4500/−4500 V; temperature—550 °C; Ion Source Gas1—55 psi; Ion Source Gas2—55 psi.

### 4.6. Statistical Analysis

All data were subjected to statistical analysis using SPSS 20.0. Analysis of variance (ANOVA) was performed on the datasets. Data are presented as the mean ± standard deviation (SD) from three independent biological experiments. Significant differences between treatments and control were determined using one-way ANOVA followed by the Dunnett post hoc test.

## 5. Conclusions

This study revealed the commonalities and specificities in gene expression and metabolite levels related to flavonoid and carbohydrate metabolism between two wheat genotypes—Fielder (Al-tolerant and P-efficient) and Ardito (Al-sensitive and P-inefficient)—under Al and LP stress, through an integrated analysis of transcriptome and metabolome data. Specifically, we found that more responsive genes and metabolites were involved in flavonoid metabolism under LP stress in Fielder, whereas Ardito displayed an opposite trend. Additionally, both Al and LP stress reduced the expression of carbohydrate-related genes and the levels of metabolites in both wheat genotypes, although an overall upregulation was observed in Fielder compared to Ardito. This upregulation is likely one of the factors contributing to Fielder’s stronger resistance to Al and LP stress. Furthermore, numerous transcription factor-related genes, such as those from the MYB, bHLH, NAC, and AP2/ERF families, were activated in response to these stresses. The present study provides novel insights into the molecular mechanisms underlying wheat tolerance to Al and LP stress.

## Data Availability

The original contributions presented in this study are available in the article or Supplementary Materials. The RNA-seq raw data can be found on the NCBI repository, accession number PRJNA1033153.

## Supplementary Materials

The following supporting information can be downloaded at https://www.mdpi.com/article/10.3390/ijms25179273/s1.

## Abbreviation

EL	electrolyte leakage
MDA	malondialdehyde
OA	organic acid
O_2_^−^	superoxide anion
POD	peroxidase
ROS	reactive oxygen species
SOD	superoxide dismutase
TF	transcription factor
