# Supplementary figures and images for: Commonalities and Specificities in Wheat (Triticum aestivum L.) Responses to Aluminum Toxicity and Low Phosphorus Revealed by Transcriptomics and Targeted Metabolomics
Source: Int J Mol Sci. 2024 Aug 27;25(17):9273. doi: 10.3390/ijms25179273 (PMC11395158; doi:10.3390/ijms25179273)

Correlation between samples

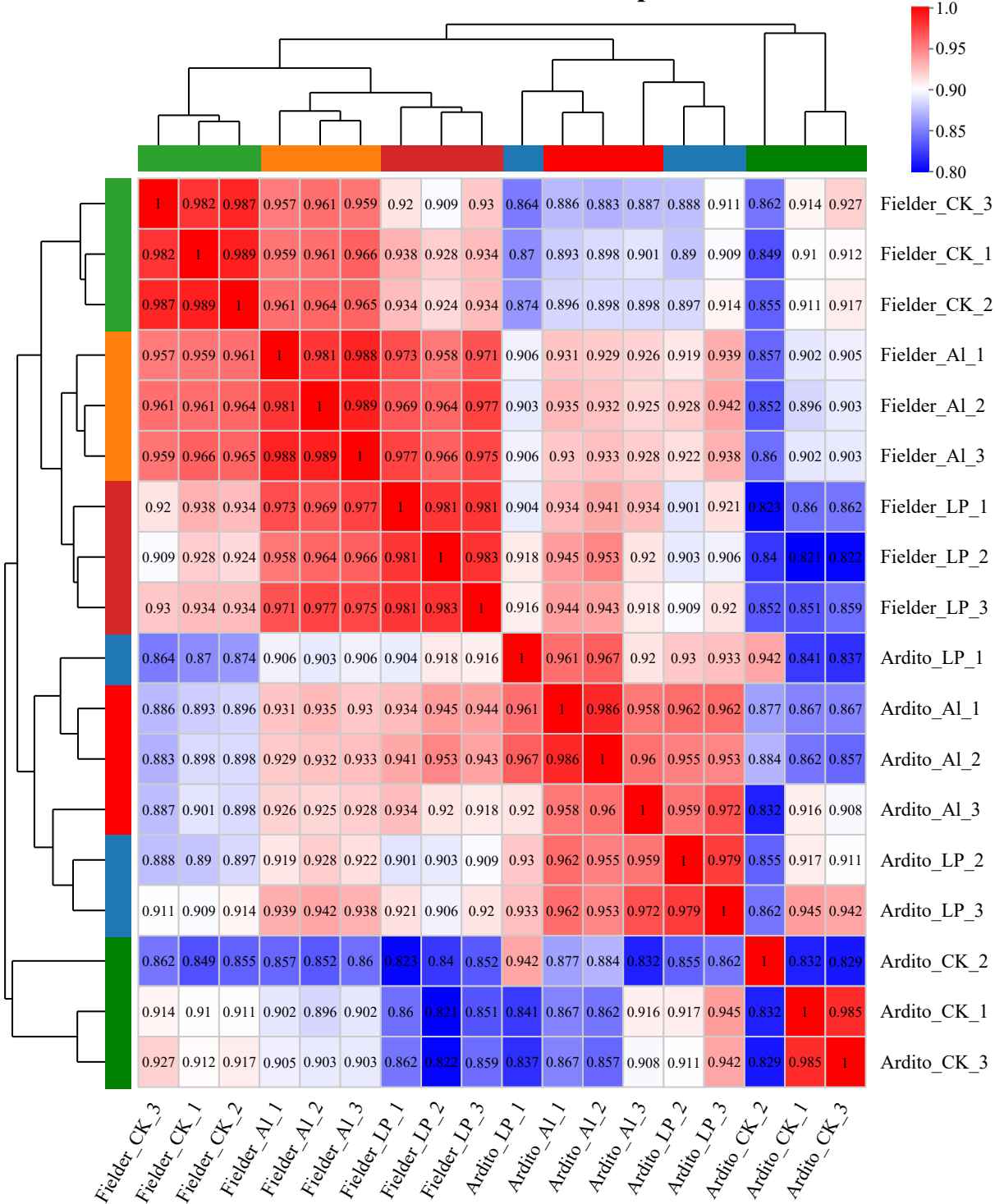

Supplement: Supplementary file 1 [file ijms-25-09273-s001.zip › Figure S1. Correlation between samples.pdf]

**$y = 0.8217x + 0.385532$**

**$R = 0.8885$**

**FC\_qRT-PCR**

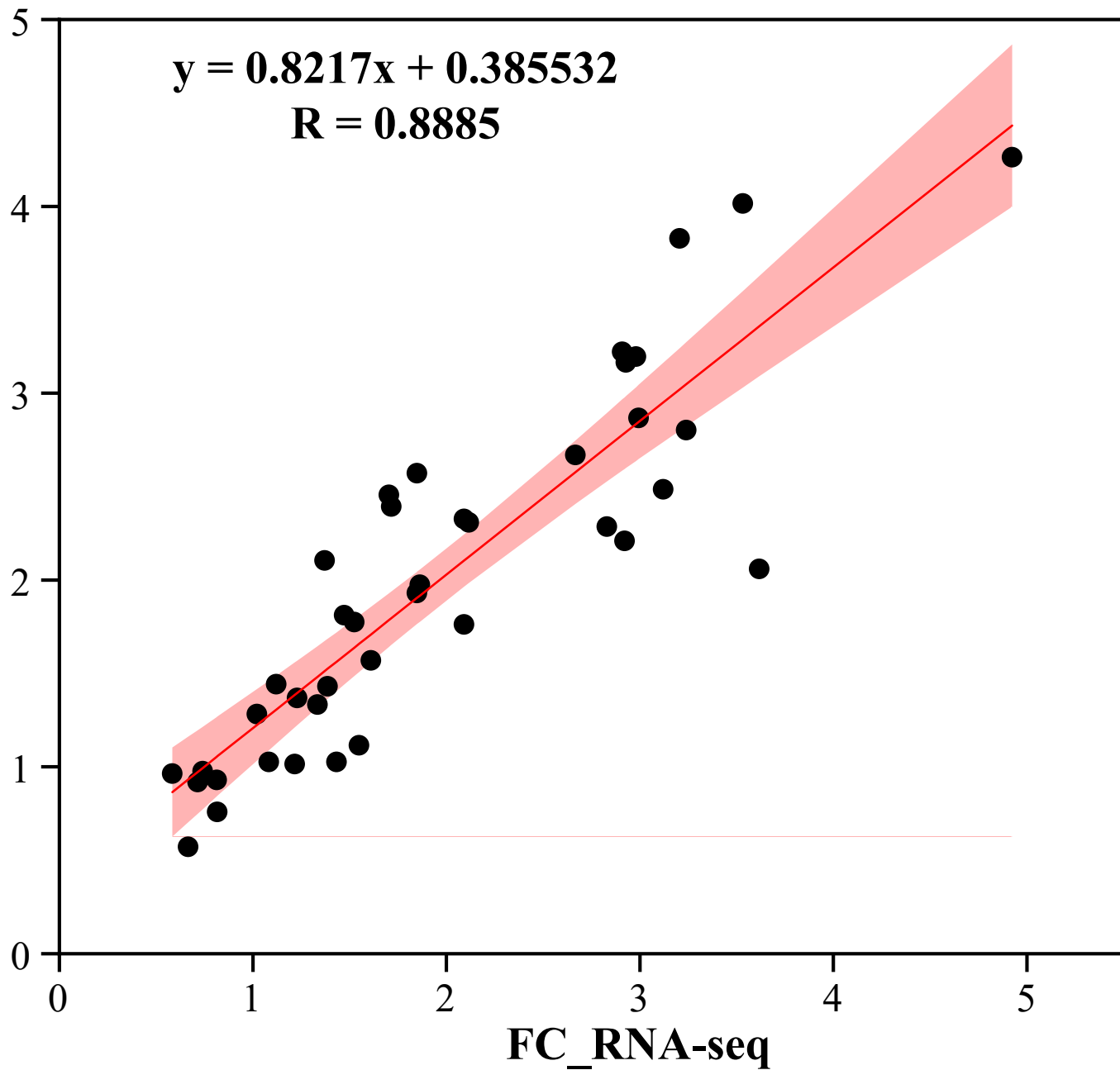

**FC\_RNA-seq**

Supplement: Supplementary file 1 [file ijms-25-09273-s001.zip › Figure S2. Correlation analusis of gene expression between Transcriptome data and qRT-PCR results.pdf]
